# Pancolitis Post COVID-19 Infection: A Case Report

**DOI:** 10.7759/cureus.31384

**Published:** 2022-11-11

**Authors:** Aymen Al-Roubaie, Ruwangi Udayasiri

**Affiliations:** 1 General Surgery, Goulburn Valley Health, Shepparton, AUS

**Keywords:** coronavirus, coronavirus disease 2019, covid -19, general and colorectal surgeon, conservative therapy, general gastroenterology, pancolitis, covid 19

## Abstract

Gastrointestinal tract (GIT) symptoms are increasingly reported as the presenting symptoms of coronavirus disease 2019 (COVID-19). These symptoms vary from diarrhea to severe colitis or bleeding. This paper reports a rare case of pancolitis as a consequence of GIT involvement secondary to active COVID-19 in a previously healthy 52-year-old lady. The diagnosis was confirmed by a CT scan of the abdomen and the patient was hospitalized and treated conservatively and discharged home after three days of hospital admission. She was followed up in the outpatient surgical clinic in two weeks with no more gastrointestinal symptoms and a normal physical examination. Careful consideration of gastrointestinal symptoms in the context of COVID-19 and a prompt diagnosis will facilitate early recognition and management and avoid any sinister complications.

## Introduction

Coronavirus disease 2019 (COVID-19) is a pandemic disease caused by the SARS-CoV-2 virus. While pulmonary symptoms like cough and shortness of breath are predominant, gastrointestinal tract (GIT) symptoms are increasingly described and reported in papers. These symptoms vary from diarrhea to severe colitis, bleeding, or perforation [1]. Colitis is defined as inflammation of the whole large bowel and it is attributed to multiple etiological factors such as ischemia, infection, neutropenia, and inflammatory bowel disease (ulcerative colitis and Crohn's disease) [2].

GIT involvement is attributed to viral direct invasion into intestinal tract cells in addition to immune host reaction [3].

We report a case of pancolitis secondary to active COVID-19. To the best of our knowledge, only three cases of pancolitis due to COVID-19 have been published.

## Case presentation

**Figure 1:**
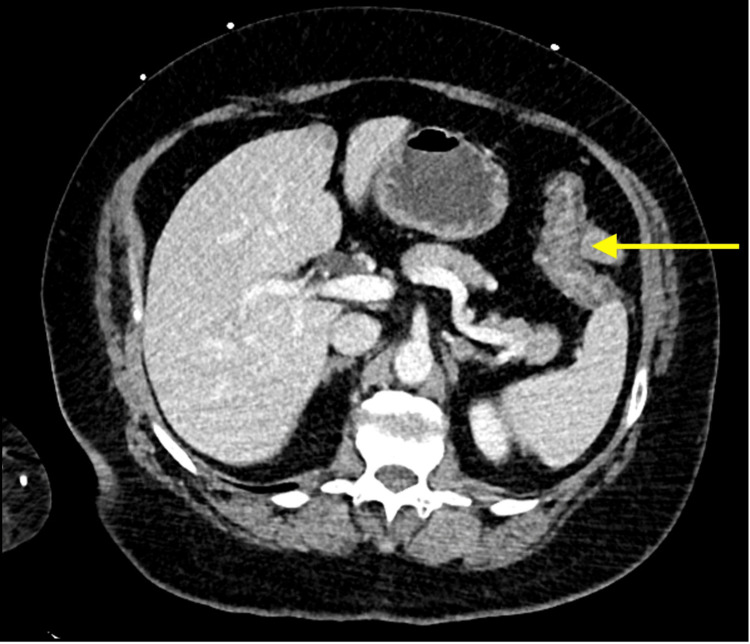
CT scan showed thickened colonic wall (yellow arrow)

**Figure 2:**
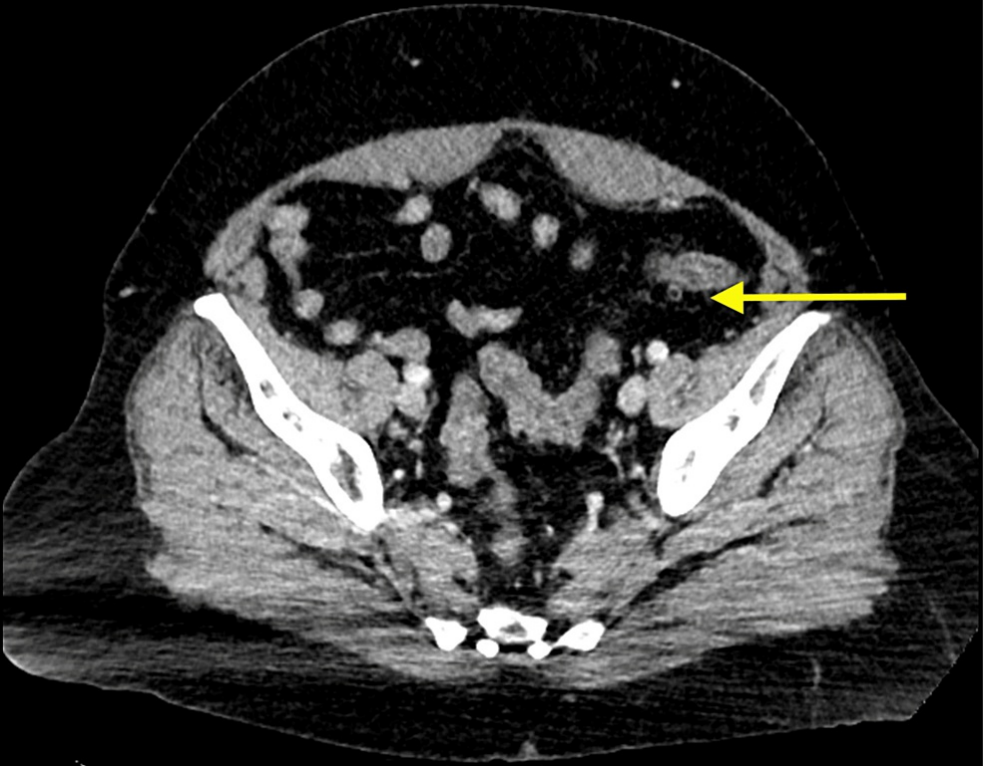
CT scan showed thickened colonic wall with mild fat stranding (yellow arrow)

A 52-year-old lady presented to the emergency department (ED) at a regional hospital with a mild cough and low-grade fever for two days. A screening test in the form of a rapid antigen test was negative and the patient was treated symptomatically and discharged home. However, the patient reported worsening symptoms such as cough and mild shortness of breath, so a diagnostic polymerase chain reaction (PCR) test for the SARS-CoV-2 virus was done and reported positive for COVID-19. The patient was hemodynamically stable and as per the COVID-19 policy, she was treated with oral antiviral in the form of nirmatrelvir and home isolation. On day four of isolation at home, the patient developed abdominal pain, colicky in nature, followed by vomiting and diarrhea, which was watery and four to five times in frequency, with a mild temperature of 37.8°C. Neither blood nor mucous was noted in the stool. No contact with a sick patient or who has similar symptoms was identified. She otherwise was healthy with no previous history of inflammatory bowel disease as well as unremarkable family history. The patient represented to the ED due to the worsening of her symptoms, as the abdominal pain worsened in severity and frequency associated with vomiting and loss of appetite. On examination, she was mildly dehydrated with vital signs within normal range. Her abdominal examination revealed mild tenderness all over but mainly left iliac fossa with no signs of guarding or peritonism. Her serum C-reactive protein was elevated at 14 mg/L (normal < 5 mg/L) and a white cell count was normal. Her other parameters of significance including hemoglobin, liver function test, lipase, renal function test, lactate, and troponin were within normal range. Abdominal CT scan showed acute diffuse colonic wall thickening and edema suggestive of an acute pancolitis with no perforation or free gas and normal other abdominal organs (Figures 1, 2). Therefore, the patient was hospitalized and treated with bowel rest and supportive intravenous fluid. No antibiotics were given and serial abdominal examinations and daily blood tests were done. The stool culture was negative, and the patient was discharged on day three after her abdominal pain improved and she was able to tolerate oral fluid and retained normal bowel habits. She was followed up in the outpatient surgical clinic in two weeks with no symptoms and a physical examination showed a soft non-tender abdomen with no signs of peritonism.

## Discussion

Gastrointestinal involvement is a recognizable manifestation of COVID-19. It has been attributed to the invasion of the virus into the bowel wall, with variable presentations from mild diarrhea to more severe forms like colitis [4-9]. Angiotensin-converting enzyme 2 receptors and transmembrane serine protease are highly expressed in gastrointestinal epithelial cells and provide a favorable route for viral entrance [10]. Gastrointestinal involvement is attributed to direct viral attack and inflammatory response secondary to the activation of inflammatory cytokines with subsequent activation of anti-inflammatory or protective cytokines [3]. SARS-CoV-2 viral RNA has been encountered in the stool samples from infected patients [11] in addition to the detection of SARS-CoV-2 RNA in tissue histology from infected individuals [7].

Acute colitis can be caused by multiple etiological factors such as inflammatory bowel disease, infection, antibiotics administration, low blood supply [12], and recently COVID-19 virus infection [4].

Pan colitis can be diagnosed with CT imaging [13] and treated symptomatically unless signs of perforation or complication have been encountered.

The index case has no previous history of inflammatory bowel disease and has not been on any medications prior to her presentation to our health facility. She has been managed with bowel rest, intravenous fluid, frequent monitoring of vital signs, and abdominal examination monitoring and was discharged from the hospital on day three after improving her gastrointestinal symptoms. Another researcher has reported bowel perforation as a complication of pancolitis [14], while our case recovered with conservative treatment. She was followed up in our outpatient surgical clinic with no relapse of her symptoms and a normal physical examination, which suggest COVID-19 as the most likely etiological factor of her colitis.

## Conclusions

COVID-19 is a multisystemic disease and gastrointestinal systems involvement has risen worldwide. Pancolitis post-COVID-19 is a rare but drastic consequence. Therefore, careful consideration of patient presentation and a prompt diagnosis will facilitate early recognition and management and avoid any sinister complications.
